# How institutional forces, ideas and actors shaped population health planning in Australian regional primary health care organisations

**DOI:** 10.1186/s12889-018-5273-4

**Published:** 2018-03-20

**Authors:** Sara Javanparast, Toby Freeman, Fran Baum, Ronald Labonté, Anna Ziersch, Tamara Mackean, Richard Reed, David Sanders

**Affiliations:** 1 Southgate Institute for Health, Society and Equity/Flinders University, Adelaide, Australia; 2 School of Epidemiology, Public Health and Preventive Medicine, University of Ottawa, Ottawa, Canada; 3 Discipline of General Practice, Flinders University Richard Reed, Adelaide, Australia; 4 School of Public Health, University of the Western Cape, Cape Town, South Africa

**Keywords:** Health systems, Primary health care, Population health planning, Institutional theory, Bio-medical model of care

## Abstract

**Background:**

Worldwide, there are competing norms driving health system changes and reorganisation. One such norm is that of health systems’ responsibilities for population health as distinct from a focus on clinical services. In this paper we report on a case study of population health planning in Australian primary health care (PHC) organisations (Medicare Locals, 2011–2015). Drawing on institutional theory, we describe how institutional forces, ideas and actors shaped such planning.

**Methods:**

We reviewed the planning documents of the 61 Medicare Locals and rated population health activities in each Medicare Local. We also conducted an online survey and 50 interviews with Medicare Local senior staff, and an interview and focus group with Federal Department of Health staff.

**Results:**

Despite policy emphasis on population health, Medicare Locals reported higher levels of effort and capacity in providing clinical services. Health promotion and social determinants of health activities were undertaken on an ad hoc basis. Regulatory conditions imposed by the federal government including funding priorities and time schedules, were the predominant forces constraining population health planning. In some Medicare Locals, this was in conflict with the normative values and what Medicare Locals felt ought to be done. The alignment between the governmental and the cultural-cognitive forces of a narrow biomedical approach privileged clinical practice and ascribed less legitimacy to action on social determinants of health. Our study also shed light on the range of PHC actors and how their agency influenced Medicare Locals’ performance in population health. The presence of senior staff or community boards with a strong commitment to population health were important in directing action towards population health and equity.

**Conclusions:**

There are numerous institutional, normative and cultural factors influencing population health planning. The experience of Australian Medicare Locals highlights the difficulties of planning in such a way that the impact of the social determinants on health and health equity are taken into account. The policy environment favours a focus on clinical services to the detriment of health promotion informed by a social determinants focus.

**Electronic supplementary material:**

The online version of this article (10.1186/s12889-018-5273-4) contains supplementary material, which is available to authorized users.

## Background

Health systems worldwide are experiencing frequent reorganisation. As this occurs, there are competing norms driving change [1]. One such norm is that of health systems’ responsibilities for population health and health equity as distinct from a focus on the provision of clinical services [2]. A population health approach considers the socio-economic conditions that play a powerful determining role in both health and equity in health outcomes. At the same time, health systems are under fiscal pressure to increase efficiency and contain costs [3]. These often-competing imperatives are central to the decisions actors in the health system have to make. In this paper, we report on a case study of population health planning in Australia occurring within ongoing reforms in primary health care. Using institutional theory, we describe how institutional factors, ideas and actors conditioned and constrained such planning. We conclude with a brief discussion of transferable lessons from this case study for population health planning processes in other health systems.

The term *population health* refers to actions directed towards improving the health of an entire population [4]. It has also come to reflect a social model of health that is concerned not only with aggregate health improvements, but also with their equitable distribution across population groups [4]. This distribution results from individual and population policies and interventions that affect socio-economic determinants of health [5]. In this way population health gives explicit attention to the growing evidence base on the social conditions that create, maintain or diminish health in individuals and populations [6, 7].

A population health approach to public health planning shifts attention away from a main focus on treating disease to one also concerned with the socio-economic causes of ill health. For example the adoption of a population health and equity lens in all domains of policy, programs, interventions and services to improve health equity is emphasised in the Victorian Healthcare Association of Australia’s planning document [8, 9]. Keleher [10] states that a social model of health including actions on social determinants of health is a key point for the best practice population health planning. Examples of high-level adoption of this approach include the World Health Report 2000, which explicitly defines the health system’s role as ‘improving health and health equity’ [2]. As stressed by the World Health Organization’s Commission on Social Determinants of Health, evidence-informed strategies such as inter-sectoral actions on health determinants, strong involvement of community-based organisations and engagement of citizens in health planning decision and actions, redistribution of funding and resources towards population groups with greater health needs, and revitalising comprehensive primary health care (PHC) needs to be fully implemented to improve health equity [11, 12]. Although this broad interpretation of population health has been widely accepted, it remains marginal in policy making processes that continue to be dominated by medicalised and individualised paradigms [13]. In many countries, regional PHC organisations have been established to plan and coordinate regional PHC services. For example, Primary Health Care Trusts in the UK established in 2002 aimed to improve PHC through service integration and community engagement. However, the UK Coalition Government abolished these PHC Trusts in 2013 and ‘devolved power and responsibility for commissioning services to local consortia of GP practices’ [14]. New Zealand’s PHCOs established in 2001 as part of the country’s PHC strategy [15] and PHC organisations in some Canadian provinces, such as Alberta and Ontario [16] provide other examples of regional PHC organisations with the primary aim of regional planning for population health and service coordination. The Canadian Community Health Centre organisation has provided some evidence on the role of PHC centres in addressing social determinants of health through local community initiatives [17].

### Australian PHC system and population health planning

The tension between biomedical and social models of health has long been noted globally [18, 19] and still remains central in Australian health policy [18]. The Australian community health centres and Aboriginal Community Controlled Health Organisations (ACCHOs) have a long history of PHC planning and practice based on a social model of health and equity [20, 21], while much fee-for-service General Practice is firmly grounded in a medical model of practice. In recent years, an emphasis on strengthening PHC to address the rapid growth in the burden of chronic diseases, an ageing population, and persistent health inequities between Indigenous and non-Indigenous people has been the focus of health reforms in Australia. The Australian National Primary Health Care Strategy published in 2009 [22] took a broad view of health and focused on equity, chronic disease prevention, and social determinants of health as key priorities.

The first Regional PHC Organisations, 116 Divisions of General Practice led by General Practitioners (GPs), were established in the early 1990s, funded by the Australian Government with a very strong focus on supporting general practitioners and general medical practice. In 2011, the Australian Labor Government, in response to the National Primary Health Care Strategy, established a network of 61 ‘Medicare Locals’ to replace the GP divisions and broaden the emphasis from supporting General Practice to a larger emphasis on population health and primary health care. Medicare Locals covered specific geographical areas across Australia and varied in the size of population they supported. Medicare Locals were funded to plan and deliver programs either directly or by commissioning on five key objectives established by the federal government:

- improving the patient journey through developing integrated and coordinated services;
- supporting the clinicians and service providers;
- identifying and responding to local health needs;
- facilitating the implementation of primary health care initiatives and programs, and;
- operating under a strong governance and effective management framework [23].

Prior to completion of the initial three year funding cycle, Medicare Locals’ effectiveness was reviewed and critiqued by the incoming government. As a result of the review, Medicare Locals were replaced by new primary health care organisation named ‘Primary Health Networks’ [24]. These organisations represent the latest iteration in the history of Australian regional PHC organisations, responsible for planning PHC to improve service integration.

### Theoretical framework

Our study is framed by institutional theory, which examines the role of, and interactions between institutions, actors and ideas in shaping policy and practice [25]. Scott, an organisational sociologist, defines institutions as ‘social structures that have attained [a] high degree of resilience [and are] composed of regulative, normative, and cultural-cognitive elements that, together with associated activities and resources, provide stability and meaning to social life’ [26]. The regulative element deals with policies, rule-setting and legal obligations with coercive mechanisms acting as the key driving force for action, meaning ‘organisations act in a certain way because they *have to’*. The normative element is concerned with values and norms which define goals or objectives, in other words ‘assumptions and expectations about what *ought to* happen’ [27]. The cultural-cognitive element stresses conceptions of the nature of social reality and the frames through which *meaning* is made. Scott states that although situations may arise where one or another element is predominant and undermines the effects of the others, they often act in combination [28]. In addition to the three key elements (regulative, normative and cultural-cognitive) driving organisational behaviour, *actors* and their *agency* are seen as crucial in influencing organisations’ behaviour, including planning and practice [29–31]. According to Scott [27] actors include: a) single individuals, or associations/population of individuals and/or; b) organisations, or groups/associations of organisations . *Ideas* are defined as ‘knowledge or beliefs about what is (evidence-base), what ought to be (values) or a combination of the two’ and are closely linked with the normative pillar [31].

Key institutional theory concepts used in our analysis of data are defined in Table 1.

**Table 1:** Key institutional theory concepts applied to Australian Medicare Locals

Institutions:
- Regulative -refers to the PHC policy context, rule-setting and legal and contractual obligations between Medicare Locals and the funding body (federal government) that impact on the structure and activities of Medicare Locals around population health planning
- Normative -refers to organisational norms and feeling of social obligations - what they ought to do – that are morally govern and underpin actions in Medicare Locals
- Cultural-cognitive - refers to the common beliefs and logics of action that are taken for granted. The social model versus medical/clinical approaches to health service delivery is considered as a cultural-cognitive element impacting on Medicare Locals’ population health planning
Actors and agency:
Refers to all the key players within the PHC institutional field and their ability to pursue decisions that can move the organisation in a new direction. Medicare Local staff are key actors. Medicare Locals’ organisational capacity and leadership can enable or constrain them to employ a broader population health approach. Other PHC organisations such as state departments of health, non-government organisations, community-based organisations, and professional associations also shape the way population health is planned and implemented.
Ideas:
Refers to the values around PHC among people at all levels of policy and practice that impacted on the ways Medicare Locals framed population health and equity.

## Methods

As part of a 3 year project we examined the population health planning processes used by Medicare Locals. We reviewed the planning documents of every Medicare Local in Australia. We also collected primary data through an online survey of Medicare Locals’ staff, individual interviews with Medicare Locals’ senior staff and a focus group and interview with staff from the federal department of health. Ethics approval was granted by the Flinders University Social and Behavioural Research Ethics Committee.

### Review of Medicare Locals’ population health planning documents

Documents including comprehensive needs assessment completed in 2014 (available for 58/61 Medicare Locals, 95%), annual plans (40/61, 66%), and annual reports (54/62, 88%) from the 61 Medicare Locals were reviewed to examine key objectives and strategies, and the extent to which they incorporated principles of population health into plans and strategies for action. Documents were publicly available and collated from Medicare Local websites. Medicare Locals had between 1 and 3 annual plans and annual reports depending on when they commenced operation. Plans and reports for 2012–13 and 2013–14 that most Medicare Locals had completed were selected for review. Collated documents were then transferred to QSR NVivo software. Adopting the WHO report characterising health systems oriented towards population health [32] and the report on the key elements of a population health approach produced by Health Canada [33] we coded the documents according to the following elements to define good practice population health planning in Medicare Locals: governance and leadership supportive of a population health approach; organisational capacity; partnerships with PHC stakeholders (in the case of Medicare Locals, with state and regional departments of health and non-government organisations); consideration of health equity in identifying needs and planning (both equity of access to health services and equity in health outcomes); mechanisms for community engagement; planning and fiscal support for health promotion; and action on social determinants of health.

Each domain was rated from 1 to 10 indicating weak, moderate and strong performance against the key elements of good practice in population health planning, depicted using a ‘traffic light’ colouring system: Score 8–10 (green – strong evidence), 4–7 (amber – some evidence), and 1–3 (red – no evidence) were given against each of the population health planning elements (see Additional file 1). Two members of the research team double coded documents from three Medicare Locals and regularly met to discuss the coding framework and emerging themes.

### Online survey of Medicare Locals’ senior members

An online survey of senior staff and board members from Medicare Locals was conducted (using SurveyMonkey) between September and November 2014. The survey instrument was developed and refined in a series of team meeting discussions and piloted with three individuals from different Medicare Locals. The survey included quantitative and open ended questions on Medicare Locals’ key achievements, engagement strategies, and the organisations’ effort and capacity in relation to population health activities. Study information and the link to the online survey were sent to the CEOs of 61 Medicare Locals for completion and distribution among senior staff including Deputy CEOs, senior executives, Board members and program managers. We used the Dillman [34] method to increase the response rate by sending an advance notification letter to the CEOs providing project information, followed by an email containing the survey link, and three follow up emails in three week intervals. We received 210 survey responses from 52 Medicare Locals (85% of Medicare Locals).

### Telephone interview of Medicare Locals’ senior staff

Survey participants were provided with the option of including their details for a follow up interview, with 106 people (50%) indicating their willingness to do so. The final selection of interview participants was based on their seniority and involvement in population health planning, and an effort to ensure geographical diversity (e.g. both urban and rural Medicare Locals). Fifty-one people were invited, with one person declining due to a change in role. Semi-structured interviews were conducted with the remaining 50 people between November 2014 and Feb 2015. Interviews focused on factors enabling or constraining population health planning, governance and decision making processes, partnership with stakeholders and community engagement. Interviews were audio-recorded, transcribed and de-identified for further analysis. Qualitative thematic analysis [35] was undertaken using QSR NVivo software. A coding framework was developed based on key concepts from the research questions and discussed during research team meetings. Eight interviews were double coded by members of the team and discussed in an analysis workshop to ensure rigour of data analysis and interpretation.

### Telephone interview and focus group with federal department of health staff

One telephone interview and one focus group discussion with four participants were conducted with senior staff at the federal department of health who had responsibility for Medicare Local policies. The focus group was facilitated by two members of the research team. The discussion points included their perceptions of the role of PHC organisations in population health planning, addressing equity and social determinants of health, and supports that are offered by the federal government to assist PHC organisations (including past Medicare Locals and newly established Primary Health Networks).

#### Data analysis and synthesis

We developed an analysis and coding framework for qualitative data including documents and interviews data. Analysis process was regularly discussed and revised during project team meetings. This process assisted to strengthen the analysis process and data interpretation, and to compare and contrast data from different sources [36]. Data were triangulated where findings from various data sources were consistent [37]. This helped to promote validity and to ensure the interpretive synthesis of data was rigorous, theory-based and a policy-relevant narrative of key factors influencing population health planning in PHC organisations.

## Results

We report on Medicare Locals’ performance in different elements of population health planning activities. Drawing on the institutional theoretical framework, we also present findings in relation to factors that enabled or constrained Medicare Locals’ population health planning. Table 2 summarises the data from different sources in relation to conceptual elements of theoretical framework underpinning this study.

**Table 2:** Data sources in relation to conceptual elements of theoretical framework

Conceptual elements of the institutional theory underpinning data analysis and synthesis	Strength of evidence from different data sources			Key finding from the data sources
	Document review	Online survey	Interviews	
Regulatory context (policies, rule settings and contractual obligations) impacting on the structure and activities of MLs around population health planning	✓✓✓	✓	✓✓	- Centralised control of programs, funding and priorities, the lack of autonomy and flexibility, short timeframes, and little recognition of health promotion and social determinants. These factors limited MLs’ capacity for population health planning and were strongly evident from the MLs guidelines and documents and confirmed in data from survey and interviews. A positive findings was the Federal policy emphasis on the inclusion of broader PHC professionals in MLs’ governance to incorporate the perspective of the wider PHC community beyond general practitioners.
Normative context (organisational norms, values, feelings of social obligations in relation to population health planning	✓	✓✓	✓✓✓	- Mixed views on social determinants of health: there were efforts by some MLs to implement strategies e.g. collaboration with state government and community organisations, using flexible funding and establishment of inclusive governance to overcome regulatory barriers to doing health promotion and social determinants of health. Examples of alignment between regulatory and normative forces e.g. partnership with the state departments of health as an enabling factor for population health planning.
Cultural-cognitive context (common beliefs and logics of action taken for granted e.g. social model versus medical approach)	✓✓	✓✓	✓✓✓	- Biomedical, service delivery approach most evident and very little attention to broader determinants. This approach was promoted by Federal government policy and guidelines and confirmed through survey and interview data
Key actors the their ability to pursue decisions in population health planning	✓✓	✓✓✓	✓✓✓	- State department of health, public and private health providers and professionals were reported as key actors in MLs’ planning process. Community involvement limited to consultation and information sharing. Little evidence of the involvement of actors outside health (including local government and social sectors) in population health planning. Although documentary sources included a range of organisations that MLs worked with, the survey and interview data revealed their limited contribution to decision making and PHC planning.
Ideas (values around PHC among PHC stakeholders)	✓	✓	✓✓✓	Conflicting ideas on concepts associated with population health, health equity, health promotion and addressing social determinants of health, as collected from different sources particularly interviews assisted to provide information about existing values around PHC and it variation among stakeholders.

### Medicare Locals’ planning for population health

**Fig. 1:**
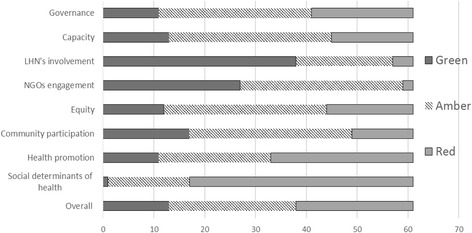
The performance of Medicare Locals against the key elements of population health planning

Medicare Locals, as mandated, put a high level of effort in identifying needs and developing population health plans, with 77% of survey respondents across the sample rating their effort in population health planning ‘high’ or ‘very high’. Needs assessment and population health planning were the most commonly cited achievements reported by survey respondents. The process of needs assessment provided them an opportunity to collaborate with state and regional departments of health, local governments, non-government organisations, public and private health providers, and to engage with local communities. The traffic light analysis of the planning documents and reports shows how Medicare Locals, in general, performed against the key elements of population health planning (Fig. 1).

### Governance and capacity

Performance in governance support and organisational capacity for population health planning was mixed with only 11 Medicare Locals documenting strong public and population health expertise in their board. When examining all the data, Medicare Locals with stronger governance i.e. community representation on their board, active engagement with community members and organisations, and equity focused strategies tended to be more likely to have higher scores in relation to implementing a population health approach including investment in health promotion activities and social determinants of health. An example was the presence of a board member with longstanding experience in health promotion in one Medicare Locals that facilitated comprehensive work on enhancing the capacity and action on health promotion.

*We have health promotion personnel working with each of the local government, the hospital sector, the community health sector who come together and do work together on priority areas that are common across the catchment. That addresses the health literacy issues…oral health for children, promoting good oral health, nutrition, those areas that are common need areas, priority areas, physical activity, we get very involved with. (CEO interview).*

### Partnership

**Fig. 2:**
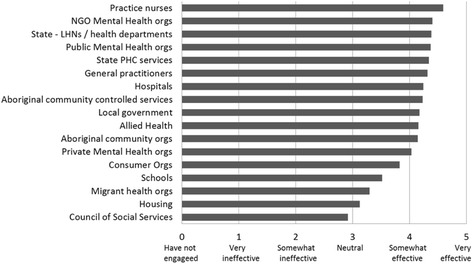
Medicare Locals’ engagement with key actors in the region

As shown in Fig. 1, the document analysis indicated that Medicare Locals implemented successful strategies to build partnerships with other PHC organisations and individual groups including general practitioners, state/territory departments of health and their regional structures (Local Health Networks, LHNs) and non-government organisations (NGOs) particularly in the area of mental health. The effectiveness of Medicare Locals’ engagement with different PHC actors and organisations is shown in Fig. 2. Eighty-two percent of survey respondents reported ‘somewhat’ or ‘very’ effective collaboration with actors in state health departments. Sixty percent of survey respondents (*n* = 127) provided examples of how they had engaged with PHC stakeholders, for example, general practitioners and LHNs. Annual reports of activities supported the survey data on the time and effort that Medicare Locals put into working with and supporting general practitioners, but mainly in areas of clinical service provision such as access to after-hour GP services, e-health, immunisation and professional training activities.

Partnership with state departments of health however varied depending on the PHC context in each jurisdiction. For example, in Victoria the Primary Care Partners (PCPs) – the state’s government-funded agency responsible for population health planning – and community health centres play an important role in PHC planning and health promotion activities and provided an opportunity for collaboration with Medicare Locals. This is cited by a Medicare Local CEO in Victoria as:

*Being that they [PCPs] are mandated to take a population health approach and use the social determinants, it broadened the scope of what our work represented. It also provided us with more resources but most importantly, it provided a common language and base for all of the health promotion and disease prevention work.*

The South Australian Department of Health, on the other hand, had experienced a major policy shift away from health promotion and population health since early 2013 [38]. This state shift provided minimal opportunities for South Australian Medicare Locals for joint planning, partnership or co-funding initiatives with LHNs.

Partnership with non-health organisations such as schools, social services and housing were rated as less effective, which aligns with the lower reported effort on addressing social determinants of health. There was an acknowledgment by some Medicare Locals’ senior staff that:

*Despite the commonality of lived experience, social determinants of health, and population overlap, there has been minimal focus on connecting social sector providers with health sector providers. (Senior executive interview).*

### Equity

All Medicare Locals collected equity-related data as part of their needs assessment. This includes mapping populations experiencing disadvantage such as Aboriginal and Torres Strait Islander people, people with non-English background and homeless people. Thirty-seven Medicare Locals mentioned equity as one of the organisational goals and/or objectives. However, strategic plans and annual reports showed that Medicare Locals focused largely on equity of access to PHC services, availability and service affordability for disadvantaged groups. Twelve out of 61 Medicare Locals considered strategies beyond equity of access by implementing equity-sensitive community based and health promotion activities. These Medicare Locals were clearly those with actors in their governance structures that had expertise in population health.

### Community participation

The needs assessment report template developed by the Federal Department of Health recommended that all Medicare Locals undertake ‘community consultation and the use of the most appropriate engagement methods’. Community engagement strategies varied amongst Medicare Locals ranging from provision of information, to more comprehensive engagement strategies. The document review indicated that all Medicare Locals used community surveys, forums and/or focus groups to seek community views on health needs and service gaps. Some Medicare Locals employed more structural community engagement strategies such as community advisory committees (27 out of 61 Medicare Locals, 44%) or representation of community organisations and members on their board (18 Medicare Locals, 29%). A few Medicare Locals had dedicated positions or teams for community engagement and a few had developed a separate community engagement strategy document.

Survey respondents were generally satisfied with the extent to which they had engaged with communities in decision making processes, with 45% (*n* = 92/208) rating community involvement as occurring ‘to a large’ or ‘very large’ extent. Community surveys and forums around health issues and access to health services were the most common examples of community engagement provided by survey respondents. In-depth interviews with Medicare Local staff suggested that community advisory committees significantly improved community inputs in decision making by providing advice to the board, although some concerns were raised around the alignment of community priorities with broader policy priorities:

*They’re providing advice and that’s great because it’s very useful advice, but we have a responsibility to look across the health system to say how does that fit into the bigger picture? (Senior executive interview).*

### Health promotion and social determinants of health

**Fig. 3:**
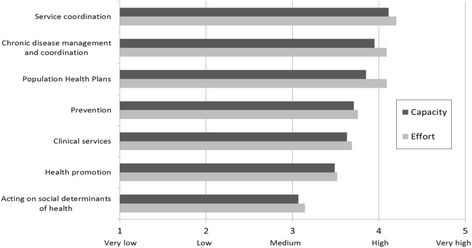
Medicare Locals survey: Effort and capacity in population health planning activities

Health promotion and social determinants of health attracted much less attention and action than the provision of clinical services (Figs. 1, 2 and 3). Federal funding allocated to specific programs such as after-hours access to GP services, access to psychological services and allied health professionals for people with mental health needs, a care coordination program for Indigenous people with chronic disease conditions and E-health (an electronic health record system containing patients’ healthcare interactions and treatments to facilitate health providers’ access to clinical information) are examples of programs focusing on clinical services that Medicare Locals were mandated to implement. Although these programs provided some opportunities and flexibilities to consider equity, e.g. targeting low income or other population groups most in need, for free or low cost services, the key focus was on frontline clinical service delivery.

The majority of Medicare Locals gathered socio-economic data including income, education, employment, housing and transport as part of their regional population profiling and recognised the link between socio-economic status of specific population groups and their poor health outcomes. The effort and organisational capacity for addressing social determinants of health was rated medium by the survey respondents (Fig. 3) reflecting their work in identifying social influences while undertaking health needs assessment. Nevertheless actions on social determinants of health were limited with only a few Medicare Locals with additional resources or strong local partnerships able to undertake local inter-sectoral action and health promotion initiatives. For 44 Medicare Locals (72%), their planning documents and annual plans indicated no action in addressing social determinants of health.

The following sections provide an analysis of the forces driving decision making and practice in Medicare Locals in relation to population health and analyses why some Medicare Local performed better than others. Using the key concepts from institutional theory we looked at the regulatory, normative and cultural-cognitive forces within the Medicare Locals institutional field, PHC actors and their interactions.

### Institutional forces and planning for population health

#### Regulatory forces

The completion of a regional needs assessment was a federal government requirement for all Medicare Locals. This needs assessment was to inform priority setting, population health planning and health service development strategies. Our findings suggest that this process was significantly constrained by the regulatory frameworks set up by the federal government and centralised control of programs, funding and priorities. This was described by participants as ‘*micro-management’*, ‘*very prescriptive’*, *‘kind of take it or leave it approach’*, and ‘*rigid* pro forma*’* from the Federal Department of Health on the way they could set priorities and implement action. A Medicare Local CEO commented: *They [federal government] have got horribly close in this process, they [population health plans] were very strongly driven by government.*

Contractual obligations and the lack of autonomy and flexibility were a common point emerging from the survey and interview data and suggests a conflict between what Medicare Locals were compelled to do and what they felt were important for improved community health outcomes:

*The major frustration is that we are contract-based. We don’t have as much freedom to do the things that we would like to do at our community with the money that we have been provided. A lot of the contracts have got very strict criteria and we feel some of those are not providing good value for money and possibly money could be spent elsewhere to get better outcomes for our community. (Chair of Board interview).*

Other regulatory forces that influenced the ability of Medicare Locals in planning for population health were the funding models, reporting requirements, and the timeframes all aligned with a predominantly curative approach as the dominant cultural-cognitive element. As one Medicare Local CEO noted:

*The funding model itself is extremely symptom… and episode of care oriented rather than person oriented. In terms of early intervention, you get the crumbs that are left over or the things that get handed out at election time.*

The unrealistic timeframes set up by the federal government for the needs assessment was a regulatory element cited as a major barrier by a majority of participants. Comprehensive needs assessment and planning involve complex processes of relationship-building with stakeholders and communities in order to collect and analyse data and translate them into actual plans. A senior executive interviewee noted:

*When I’m running a ten-week needs assessment if I haven’t got the data it’s just not going to get included... first of all having current data and then to actually have the time to analyse that more fully*. Another interview participant stated: ‘*Often we proceeded [with planning] without full population health data or provider insights…we would sometimes find that you’d be eight months into an activity and then understand that we’d miscued and that we’d made certain assumptions that were incorrect, and then we needed to modify our approach. (Senior executive, interview).*

There were however a number of regulatory factors which had a positive influence on Medicare Locals’ performance on population health. For example, the federal policy emphasising the inclusion of broader PHC professionals in Medicare Locals’ governance [23] opened a window of opportunity for most Medicare Locals to incorporate the perspectives of a wider PHC community in planning and decision making, as well as general practitioners. Some Medicare Locals with a stronger population health approach used this space more proactively to broaden their population health scope beyond general practice. Moreover, the requirement for a skills-based board facilitated inclusion of views from community members beyond those working in the health sector, such as people with legal, financial and business backgrounds. The establishment of strategic leadership groups consisting of PHC partners, community representatives, local government and community organisations in most Medicare Locals (this group was recommended in the needs assessment guide to oversee the assessment and priority setting process) was noted as a positive step in better understanding population needs and interventions. A senior executive interviewee stated:

*At the outset we established a ‘strategic leadership group’ and while that had its pros and cons, we did get some good buy in from fairly high levels at the local health network and the Aboriginal health council, and the local government was represented. We involved Health Consumers Alliance and so we had a pretty good representation who provided input in planning.*

When the Federal government changed in 2015, Medicare Locals were replaced by new primary health care organisations - Primary Health Networks, which was accompanied by some changes in regulations, and further cuts to the flexible funding pool and proposed health promotion activities and a greater emphasis on integration of primary and secondary care services and commissioning of clinical services. These changes in the regulatory environment created additional pressure and disruption to the population health planning work of Medicare Locals. There was a general consensus in our interviews that the ongoing change in PHC structure and its political nature were affecting the implementation and evaluation of population health planning negatively: *You’re creatures of a political system, the unfortunate reality is that you’re subject to the policy whims of the day. (CEO, survey)* A population health officer reaffirmed the impact of the political system of the time on population health activities:

*Population health takes time, and we constantly have to compromise in order to fit into short political cycles and to meet political imperatives rather than demonstrating health outcomes through programs with some degree of longevity. (Population health officer, survey).*

These regulatory forces contributed to shaping the normative and cultural cognitive forces affecting Medicare Locals, detailed below, to fit into these political cycles and constraints, thus affecting population health planning practices.

#### Normative forces

Normative forces are about values and what ought to be done [26]. Improving population health outcomes was a common ‘goal’ reported in 56% of Medicare Locals’ strategic plans. The importance of social determinants of health in improving population health was explicitly recognised in 9 planning documents and evident in some Medicare Locals’ interviews as noted here:

*The social determinants are very much a part of what we need to consider…health doesn’t just sit on its own. Health is influenced by the society that people live within and the conditions that they live under. (Senior executive interview).*

The conflict between normative and regulatory forces is shown in an interview with a federal policy actor who describes the views on social determinants of health as:

*While it would make logical sense to be working in those areas [health promotion and social determinants of health], obviously you broaden the scope, you broaden the requirement for resources, but you also raise the risk of this particular issue around activities that are arguably not within the scope of the constitution or head of powers. (Federal department of health focus group).*

This conflict between the normative and regulative forces constrained Medicare Locals’ planning for population health activities. As an interview participant commented: *We’ve had a very strong commitment to addressing upstream determinants of health, but we haven’t had the means to actually do that. (Chair of Board interview)* This conflict was clearly reflected in the activities documented in annual reports, and was supported by interview and survey respondent data. Fifty percent (*n* = 95) of survey respondents rated ‘policy context not supportive of population health’ and 57% (*n* = 111) rated ‘health system prioritising clinical care’, as obstacles that to a ‘large’ or ‘very large’ extent constrained their ability to plan for population health activities. One interview participant commented:

*Prioritising in the notion that you just wanted to do what the government was stipulating…it created a pessimism in the population health team because a lot of them were saying “All these important findings are there, but I don't think any of them might go into the eventual planning” because a lot of them were often health promotion and prevention. (Senior executive interview).*

Despite the lack of support from the federal government some Medicare Locals implemented strategies to overcome barriers to doing health promotion. Examples were partnering with state government or community organisations to co-fund and collaborate in local community-based programs or using flexible funding (despite funding insufficiency) to overcome the predominance of planning for clinical services:

*Now the new management team, of course, really wanted to change that [clinical predominance] but we weren’t able to. So we have gone into an arrangement with [organisation’s name], which is an NGO from that Healthy Cities movement [and] which has real expertise in health promotion. (Senior executive interview).*

Other Medicare Locals positioned their health promotion work using language consistent with regulatory priorities. As one CEO commented: *We avoided the word health promotion and [put those plans] under the stakeholder engagement of primary care support section that would be acceptable [to the federal government].* While such a strategy of circumventing regulatory constraints in health promotion work is not unusual, it has the longer term effect of legitimizing an institutional norm (clinical predominance) that is antithetical to the principles of health promotion, weakening the possibilities of future planning efforts.

This study confirms the combined strength of alignment of regulatory and normative forces. For example, partnership with the state departments of health was emphasised in the initial guidelines set up by the federal department (regulative element) and was perceived as critical to population health planning by Medicare Locals’ staff (normative element). This alignment between regulatory and normative elements seemed to be enabling for more effective population health planning and integration within PHC services and between the primary and hospital sectors.

#### Cultural-cognitive forces

The cultural-cognitive element of institutional fields provides ‘templates for framing individual and organisational perceptions and decisions’ with taken-for-grantedness as the basis of compliance [26]. Our study findings suggest that the still culturally dominant biomedical definition of health was aligned well with the regulatory controls imposed by the federal government but misaligned with the population health approach to planning that Medicare Locals were required to incorporate. Health promotion and social determinants of health were considered to ‘*go beyond the remit of a health portfolio’* as one federal participant expressed, and were being ‘*managed by different entities’*. From the perspective of a Medicare Local survey participant: *Social determinants of health appear to be off the radar (Senior executive, survey)* A population health manager further noted how the timeframes regulated by the federal government align with *‘acute care and hospital sort of health time frames rather than primary health care and population health.’ (Project manager interview).*

This cultural-cognitive force of a biomedical, service delivery approach affected Medicare Locals in different ways. This biomedical approach was accepted in some Medicare Locals that lacked explicitly stated population health norms. This allowed them to follow the regulatory forces and implement activities limited to clinical services. For other Medicare Locals, the biomedical cultural-cognitive force conflicted with a normative commitment to undertaking prevention and health promotion as key activities to improve population health and health equity.

### Differing ideas on population health and equity

Ideas on PHC, population health, health equity, and the role and contribution that the health system should play in health promotion and addressing social determinants of health interconnects with the normative and cognitive-cultural elements of the institutional field. Medicare Locals participants’ responses revealed conflicting ideas on concepts associated with population health. Health equity, for example, was generally restricted to improving access to PHC and clinical services for ‘disadvantaged’ population groups or geographical areas identified in the initial needs assessment. ‘*Improved the number of GPs available*’, ‘*delivering services in country areas’*, ‘*extending access to after-hour services’* and ‘*creation of new health services for marginalised groups*’ are examples of interventions reported by participants to improve health equity. There were only a few participants explicitly stated equity in health outcomes and the need for broader and long term interventions to achieve health equity:

*They’re two different questions. The improvement in access - yes. We’re the first group to get an Indigenous after-hours service up and running. So that’s clear, it’s tangible - great result. Have we made a difference? Well, we’ve made a small difference, are we addressing needs? No, we’re only starting, unfortunately. But the reality is, we’re looking through the prism of two years of work dealing with multi-generational issues. (Board member, interview).*

Participants’ perceptions on social determinants of health ranged from ‘*If people are poor, they’re poor, we can’t give them jobs; So we’re looking at other things and some of the issues that have come up is the level of health literacy in that local population*’, *‘they’re [social determinants of health] often beyond our control because they’re non-health related*’, to a stronger orientation towards social determinants and inter-sectoral action and its impact on health outcomes as noted by one Medicare Local CEO:

*It [SDH] has been a really core part of our focus, we very much understand that health has operated in a silo and that it can’t continue to. We need to be focused on the social care and education sectors. Every sector should work together, it’s cost effective, and it produces efficiencies. But better still it produces amazingly better health outcomes in our community, and that’s what we’re all committed to. So there’s no reason not to be doing it.’*

The differences in ideas on ‘health’ and ‘PHC’ impacted the way individual Medicare Locals performed on population health. Where Medicare Local actors were committed to a more social view of health this drove normative expectations of what Medicare Locals should be doing and provided some opportunities to achieve more in population health activities, even though this work came into conflict with the regulative and cultural-cognitive push towards a more biomedical view of health and PHC.

### PHC actors and population health planning

Our study shed light on the range of actors, within and external to Medicare Locals that play a role in Australian PHC and how their agency and interactions influenced Medicare Locals’ performance in population health planning.

Actors are argued to play a vital role in maintaining organisational values and norms, in overcoming external and regulatory pressures as described in institutional theory, and in influencing or negotiating power [39]. The role of actors and their agency provide ‘an opportunity to realise interests that they value highly’ [40]. Despite regulatory and cultural-cognitive forces constraining population health planning, in some Medicare Locals individual staff in leadership positions played an important role in pushing the Medicare Local towards a greater population health perspective and action. This is clearly shown from a senior executive interview:

*The way she [the CEO] thinks - she believes in population health, and even advocated for it to be on the senior management team when she took up the role as the CEO. Initially it was -population health reported to Corporate Services in the structure that the board had done, but the CEO said this needs to be a second tier position and to be around the executive table. There is absolutely a belief that population health and those associated concepts like equity, like the social determinants of health, need to be sitting around the centre leadership table….and then the board absolutely understood that when she was able to talk it through with them. They wholeheartedly agreed. (Senior executive, interview).*

Although Medicare Locals were mandated to establish a skills-based rather than a representative board, our study revealed examples of how the inclusion of community or Aboriginal and Torres Strait Islander representatives on boards influenced Medicare Locals’ performance on community engagement and equity. The presence of a member of the local Aboriginal Community controlled health service on the board of one Medicare Local was reported critical in ‘*effectively advocating for Aboriginal health*’ and to reduce the *‘tensions between community controlled organisations and Medicare Locals’* in the region. A review of the annual report from this Medicare Local revealed examples of *‘partnering with the community controlled Aboriginal Health Service, sub-contracting the Aboriginal services which supported additional capacity in Aboriginal programs, and co-design of a number of community based activities such working with child and family centre, healthy eating for young Aboriginal mums, and involvement in community events’*. Although the successful outcomes cannot be attributed to the individual representative on board, the inclusion of the representative on the board could be argued to have potentially fostered a greater commitment to and broadened the scope of work on Aboriginal health.

Another example was having a strong population and community engagement expert on the board that facilitated the implementation of community engagement strategies:

*Our Medicare Local Board particularly [name] is committed to consumer, carer and community participation in the planning, delivery and evaluation of the programs within our local communities so now we have a Community Participation Advisory Group that reports through to the board. The group is involved in strategic planning, consultation on new initiatives, advice on resources for the community. (Medicare Local survey).*

The ability to act/counteract federal policies was also linked to Medicare Locals’ overall capacity in population health planning. Organisational capacity is defined as ‘internal ability of an organisation to enact a specific task’ [41]. Inclusion of the concept of organisational capacity alongside other forces identified in the institutional theory has the potential to better explain uneven implementation of organisational practice or outcome measures [41]. In this study we found considerable variety in capacity for population health planning reported by Medicare Locals. While some Medicare Locals noted *‘highly capable staffing’*, and ‘*excellent expertise and skills*’ in population health planning, others, particularly rural and regional Medicare Locals, struggled to locate individuals with knowledge and expertise in collecting and interpreting population data, e.g.:

*We’re a bit under resourced and certainly at the outset it took us a while to realise that we were going to need some more people [to do population health planning] and so we had a couple of part timers really and a few people doing bits and pieces here and there. (CEO interview).*

The lack of resources, tight timeframes and poor staff training added to the challenge of employing staff or building internal capacities: *Our capacity is about resources, which we don’t have much of... (Senior executive interview).*

Those Medicare Locals with stronger population health teams and/or expertise in a specific population group showed more success in considering equity and implementing community engagement strategies. An example of organisational capacity in Aboriginal health is shown in a CEO interview:

*Our Aboriginal health team is about 50 people and 80% of those are Aboriginal people. I think that’s been useful in that, although we’re a mainstream organisation, we obviously had a lot of Aboriginal people employed and they were given valued positions and valued within the organisation and that was reflected outside into the communities as well. We gained acceptance because of that large group of Aboriginal staff that we had. (CEO interview).*

As shown in Fig. 3, organisational capacity was assessed to be much lower in health promotion and social determinants of health, reflecting a lack of focus on health promotion in the regulatory pillar, fed by the cultural-cognitive force of biomedical dominance. A senior executive noted:

*I think staff didn’t really have the skill level or knowledge base to actually broaden that scope of practice [from practice-focused activities to community-based health promotion]. I think it was around partly skill level of staff. (Senior executive interview).*

Other actors included public servants in organisations external to Medicare Locals, such as state departments of health and their regional primary and tertiary health services, and private actors, including general practitioners that could have positive or negative effects on Medicare Locals’ decision making on population health activities. The following quote shows the challenge one Medicare Local faced as a result of pressures from general practice individuals and organisations:

*At some levels of decision making, we were a bit subject to, I don’t know if you’d call it political pressure, but pressure from some outspoken general practice individuals and groups. I’m possibly being a bit ideological but I was surprised that those groups had that much influence in some of the decision making. You still need to take on board the perspectives of your stakeholders but it probably was to a greater extent than I would have expected and it was a bit unbalanced as well.’ (CEO interview).*

## Discussion

A population health approach to PHC planning and practice is essential to link the knowledge and evidence about social determinants of health with action, and to focus on service access and health outcomes using an equity lens [42]. Keleher [10] states key points for best practice in population health planning including: being based on a social model of health and health equity, recognition of determinants of health and the role of various sectors in addressing those determinants, partnership in planning, community engagement, priority setting program planning, and workforce capacity building through education and training. Our study of Medicare Locals enabled us to examine these elements of population health planning how system planning for population health was influenced by the regulative, normative and cultural-cognitive forces of the broader institutional field and interlinks between actors and their agency to determine population health planning processes and outcomes. Institutional theory provided us a framework to examine these forces, identifying the competing norms and the ways Medicare Locals operated in their efforts to improve population health.

Medicare Locals were established in response to the Australian national PHC strategy which had an emphasis on improved access, reduced inequity, and disease prevention as its key objectives [43]. Our study has shown that, despite an emphasis on regional planning, population health and local responsiveness, a narrow vision of population health emerged from the planning where little attention was paid to addressing social determinants of health and health equity. The findings from our study are consistent with the study of population health planning in the Manitoba province in Canada, which found that such planning relied mainly on ‘epidemiological evidence or population surveys and consultations as a tool for health assessment and service planning’ rather ‘than emphasising action to address social determinants of health and inequity’ [42]. Another study of PHC services in Australia has also shown that population health and health equity were given little attention [38].

In an effort to understand the relative influence of the different institutional elements on Medicare Locals’ ability in planning for population health, our study has shown the dominance and powerful influence of the regulatory forces (federal government rule setting) in directing planning processes in Medicare Locals (mainly away from population health activities). The alignment of these regulative forces with the cultural-cognitive element in the taken-for-grantedness of biomedical approaches provided further barriers for Medicare Locals in planning for population health. As noted by Scott [26], viewing institutions as ‘resting on formalised control systems and regulations’ is the dominant approach employed by economists who see institutions as setting the ‘rules of the game’. Although the obligation to undertake population health planning and to engage with community stakeholders in governance were one component of the regulatory element aligned with existing norms in many Medicare Locals, they were unable to offset the fiscal regulatory elements governing Medicare Local’s functions. Our study findings (and those of related research we have undertaken) suggest that the current pressure on health systems to increase efficiency has shifted health systems to a more centralised control and rule setting system [44]. Employment of a central regulatory system in complex health systems that are embedded in broader social systems and impacted by social determinants of health may not assist in improving population health and achieving health equity.

The second point that emerged from our study is how the normative forces (belief in what ought to be done) and ideas (commitments to a social model of health) in some Medicare Locals assisted them to push against government norms and mandates. Normative context is argued by some scholars as ‘social embeddedness of political and economic behaviour’ [45] and that decisions are responsive to not only rules and regulations but to the appropriateness of actions [46]. In response to normative forces, some Medicare Locals made efforts to develop their population health plans collaboratively through interaction with other PHC actors and by using internal capabilities that Medicare Locals had more control over, such as strengthening governance and organisational capacity in population health. Institutional theory also emphasises the importance of the role played by *actors* and their *agency* (actors’ ability to have some effect on the social world – altering the rules, relational ties, or distribution of resources) [26]. Those Medicare Locals that had strong capacity and public health advocates in their leadership, including Indigenous health or community representatives in their board were more able to develop strategies in line with the principles of population health. Our findings confirms Barman et al.’s [41] argument that examining organisational capacity enriches our understanding of the institutional theory and its explanation of uneven outcomes within different organisations.

The interactions that Medicare Locals had with PHC actors and institutions at the regional or state levels were a major factor in enabling or constraining their practice in population health. The federal-state health system divide in Australia means that different norms exist in different jurisdictions. Although creating conflict and confusion in some cases, states/territories with an emphasis on population health provided opportunities for stronger collaboration and implementation of population health activities. For example, this was the case for some Victorian Medicare Locals, enabling them to plan health promotion activities in collaboration with the state department of health.

Finally, our study has shown that the *ideas* on how population health and comprehensive primary health care are defined play an important role in policy making and in organisational performance. Dominant ideas of a biomedical service provision-oriented health system (aligned with the regulatory and cultural-cognitive elements of Medicare Locals’ institutional environment) appeared to be a constraining factor limiting upstream actions that addressed the social determinants of health.

Our study reaffirms the interaction between different institutional forces and their impact on organisational performance. Currently, the regulatory and political environment is pushing away from a social view of health, health promotion and social determinants of health. This has had a knock-on effect to Medicare Locals and constrained their ability to institute action on social determinants of health. The study of Medicare Locals’ population health planning has implications for policy and practice in the current Australian context and for the relatively newly established Primary Health Networks (Table 3). There is a need to examine strategies to foster a more conductive regulatory environment that would support policy actors to include a population health approach to PHC planning. Effective population health planning which addresses structural determinants of health would also require continuity and regulatory stability for regional PHC organisations to allow the actors a chance to plan, implement and evaluate action on social determinants, and monitor their likely impact on health equity.

**Table 3:** Policy and Practice Implications

Practice implications for PHNs’ staff and board
• Strong governance and leadership in PHC require a commitment to comprehensive PHC beyond biomedical model of care and an emphasis on health equity at population health. PHNs need to ensure the structure, composition and visions of their leadership teams support such a comprehensive vision of PHC. PHNs need to recruit staff with expertise in public and population health who understand the dynamics of population health planning – training also required for clinical staff so they gain understanding of population health approaches
• PHNs should consider the employment of strategies to maximise community participation in planning and decision making. Representation of community members on board and balancing clinical and community governance would enhance the effectiveness of population health planning in addressing health needs.
• PHNs require to formulate or built on the positive relationships with PHC stakeholders including local governments, community organisations and community-controlled health services to ensure shared goals, joint planning and resource sharing in population health. These partnerships are vitally important to improve PHNs’ chance of success in addressing population health needs and social determinants of health.
Policy implications for State government
• States require a strong comprehensive PHC policy committed to disease prevention, health promotion including action on social determinants of health and, curative and rehabilitative services in order to provide a mandate for and to foster effective partnerships and joint population health planning.
• State government staff require a sophisticated understanding of comprehensive PHC principles and the mandate and resources to work effectively with PHN staff
• State governments need to draw on existing effective collaborative partnerships to create stronger link with PHNs in planning processes.
Policy implications for Federal government
• The Federal government’s political and policy vision to PHC need to be comprehensive rather than medically-oriented. This requires a strong recognition and promotion of principles of comprehensive PHC including actions on health promotion, social determinants of health, and health equity. The commitment to PHC needs to be explicitly reflected in planning frameworks and guidelines developed for PHNs to address comprehensive PHC.
• Sufficient and flexible funding is needed to provide PHNs financial capacity and
• authority to identify and respond to local health needs.
• More investment in training of PHNs’ staff including clinical staff on population health and PHC is required to enhance the capacity and capabilities of PHNs in population planning, implementation and evaluation.
• A Federal program of response to social determinants of health is required and should be guided by the recommendations of Senate select committee [47]. PHNs could then spearhead the national health sector response to social determinants of health.

### Study strengths and limitations

The use of different data sources enabled us to identify key elements of population health planning and areas of consistency to triangulate data. This was particularly the case in examining the regulatory forces driving population health practice in Medicare Locals. In relation to documentary sources, all Medicare Locals used a similar template, provided by the Federal government, to report the process and outcomes of their comprehensive needs assessment. This helped us to compare and contrast findings and variations between different Medicare Locals in relation to population health planning and priority setting processes. Receiving survey responses from 85% of Medicare Locals ensured a good representation of Medicare Locals across Australia. The study, however, had some limitations. Firstly, as Medicare Locals commenced their operation in three tranches (19 Medicare Locals in July 2011, 18 Medicare Locals in January 2012 and 24 Medicare Locals in July 2012), the ones with latest commencement date had limited time and capacity in comparison with Medicare Locals established in the first tranche and thus limited comparability of findings. Secondly, interviews with Medicare Locals were conducted shortly before the end of Medicare Locals and introduction of Primary Health Networks. Although this have increased the willingness to participate in the interviews, this might have affected the views of participants towards a better performance of Medicare Locals on population health planning at a time of major structural change.

## Conclusion

Our study is novel in illustrating the application of institutional theory to the primary health care system which enabled us to examine the driving forces in planning and to inform analysis of how we can move towards population health and health equity oriented health systems. The study of Australian Medicare Locals provides one example of the complexity and interactions between different factors that enable or hinder planning for population health. There are numerous institutional, normative and cultural barriers to effective population health planning. The experience of Australian Medicare Locals highlights the difficulties of planning in such a way that the impact of the social determinants on health and health equity are taken into account. The political and policy environment favours a focus on clinical services to the detriment of health promotion informed by a social determinants focus.

This case study offers insights for PHC policy makers, and transferable lessons for the relatively new Australian Primary Health Networks. Study findings may also have utility for other health systems operating in similar settings, specifically on the interrelations between different institutional elements driving decisions and actions in PHC organisations, and some of the reasons why health systems, despite cycles of reform, still largely fail to adopt a population health approach as a key to improve equity in health [48].

## Additional file

Additional file 1: Traffic light scoring. Definition of key elements of the population health planning for traffic light scoring system. The table shows the definition of each element of population health planning that were used to score Medicare Locals’ activities using traffic light scoring method. (DOCX 14 kb)

## Abbreviations

ACCHOs: Aboriginal Community Controlled Health Organisations
GP: General practitioner
LHN: Local Health Networks
NGOs: Non-government organisations
NHMRC: National Health and Medical Research Council
PCPs: Primary Care Partners
PHC: Primary health care
