# Supplementary material for: How institutional forces, ideas and actors shaped population health planning in Australian regional primary health care organisations
Source: BMC Public Health. 2018 Mar 20;18:383. doi: 10.1186/s12889-018-5273-4 (PMC5861731; doi:10.1186/s12889-018-5273-4)
Supplement: Supplementary file 1 — Traffic light scoring. Definition of key elements of the population health planning for traffic light scoring system. The table shows the definition of each element of population health planning that were used to score Medicare Locals’ activities using traffic light scoring method. (DOCX 14 kb) [file 12889_2018_5273_MOESM1_ESM.docx]

**Supplement**

**Definition of key elements of the population health planning for traffic light scoring system**

|  | **Green (8-10)** | **Amber (4-7)** | **Red (1-3)** |
| --- | --- | --- | --- |
| **Governance** | Strong population health knowledge and expertise on board and executive, community representatives on board, inclusion of professions including non-medical professions | Limited population health knowledge and expertise on board and executive, community representatives on board, inclusion of professions including non-medical professions | No population health knowledge and expertise on board and executive, community representatives on board, inclusion of professions including non-medical professions |
| **Capacity** | Dedicated team and management position responsible for population health planning, strong internal capacity and skills in needs assessment and analysis | Limited capacity and skills in for population health planning, external organisations responsible for needs assessment and analysis | No capacity and skills in for population health planning |
| **LHNs’ involvement** | Strong partnership with LHNs, formal agreement, LHN representative on board, frequent meetings, joint planning, data sharing | Limited partnership with LHNs, informal agreement, occasional meetings | No evidence of partnership with LHNs |
| **NGOs’ involvement** | Strong partnership with a range of NGOs and community organisations, evidence of formal agreements, representation on board | Limited partnership with NGOs and community organisations, some evidence of informal relationships, no representation on board | No evidence of partnership with NGOs |
| **Equity** | Equity as an organisations goal, collection of disaggregated data on equity groups, strong evidence of interventions targeting equity in general and the three equity groups in particular | limited action to improve equity and address the health needs of the equity groups | No evidence of collecting aggregated data on equity groups, or action to improve equity |
| **Community engagement** | Strong community engagement strategies, community representation on board, community advisory committees, community feedback and reporting system | Limited community engagement strategies (only survey, forums, to collect information) | No community engagement strategies, Limited activities to provide information about ML activities |
| **Health promotion** | Strong evidence of health promotion activities, funding allocation, community works, partnership with Health promotion organisations, health promotion workforce | Limited evidence of health promotion activities and community work, no health promotion workforce | No evidence of health promotion activities and workforce |
| **Social determinants of health** | SDH identified as a priority area, strong collaboration with social sector, examples of action on SDH | Good understanding of SDH, some examples of collaboration with social sectors, limited interventions on SDH | No evidence of action on SDH |
